# Leveraging Big Data for Exploring Occupational Diseases-Related Interest at the Level of Scientific Community, Media Coverage and Novel Data Streams: The Example of Silicosis as a Pilot Study

**DOI:** 10.1371/journal.pone.0166051

**Published:** 2016-11-02

**Authors:** Nicola Luigi Bragazzi, Guglielmo Dini, Alessandra Toletone, Francesco Brigo, Paolo Durando

**Affiliations:** 1 Department of Health Sciences, Postgraduate School of Public Health, University of Genoa, Genoa, Italy; 2 Department of Health Sciences, Postgraduate School in Occupational Medicine, University of Genoa, Genoa, Italy; 3 Department of Neurology, Franz Tappeiner Hospital, Merano, Italy; 4 Department of Neurological, Biomedical, and Movement Sciences, University of Verona, Verona, Italy; 5 Occupational Medicine Unit, IRCCS AOU San Martino-IST, Genoa, Italy; New York City Department of Health and Mental Hygiene, UNITED STATES

## Abstract

**Objective:**

Silicosis is an untreatable but preventable occupational disease, caused by exposure to silica. It can progressively evolve to lung impairment, respiratory failure and death, even after exposure has ceased. However, little is known about occupational diseases-related interest at the level of scientific community, media coverage and web behavior. This article aims at filling in this gap of knowledge, taking the silicosis as a case study.

**Methods:**

We investigated silicosis-related web-activities using Google Trends (GT) for capturing the Internet behavior worldwide in the years 2004–2015. GT-generated data were, then, compared with the silicosis-related scientific production (i.e., PubMed and Google Scholar), the media coverage (i.e., Google news), the Wikipedia traffic (i.e, Wikitrends) and the usage of new media (i.e., YouTube and Twitter).

**Results:**

A peak in silicosis-related web searches was noticed in 2010–2011: interestingly, both scientific articles production and media coverage markedly increased after these years in a statistically significant way. The public interest and the level of the public engagement were witnessed by an increase in likes, comments, hashtags, and re-tweets. However, it was found that only a small fraction of the posted/uploaded material contained accurate scientific information.

**Conclusions:**

GT could be useful to assess the reaction of the public and the level of public engagement both to novel risk-factors associated to occupational diseases, and possibly related changes in disease natural history, and to the effectiveness of preventive workplace practices and legislative measures adopted to improve occupational health. Further, occupational clinicians should become aware of the topics most frequently searched by patients and proactively address these concerns during the medical examination. Institutional bodies and organisms should be more present and active in digital tools and media to disseminate and communicate scientifically accurate information. This manuscript should be intended as preliminary, exploratory communication, paving the way for further studies.

## Introduction

Silicosis, a form of pneumoconiosis, is an untreatable but preventable occupational fibrotic lung disease.[1–3] It is caused by the respiration, deposition and retention of inhalable free crystalline silicon dioxide or silica dust, and, less frequently, silicate mineral dust. Silicosis can progressively evolve to lung impairment, respiratory failure and death, even after occupational exposure has ceased.[1–3]

Usually, this process is chronic and may require up to 10 or more years to fully develop; however, acute or accelerated forms of silicosis may occur, even though less frequently.

No effective specific treatment for silicosis is available; however, comprehensive supportive care can be provided in order to slow the natural history of the disease and to improve patient’s quality of life. Further, some clinically selected patients may be considered eligible for receiving lung transplantation.[1–4]

Hazardous occupational exposures to silica dust can involve a variety of workers, including those engaged in mining, quarrying, denim sandblasting and abrasive blasting, sand and gravel screening, rock drilling and crushing, roads, highways and bridges construction, demolition and repair, pottery making, stone masonry, and tunneling operations, among others.[5] Recently, silicosis cases resulting from hazardous exposure to silica during hydrofracking of gas and oil wells or exposure to quartz surfacing materials, such as artificial quartz conglomerates, during construction and installation of engineered stone kitchens and bathrooms, have been reported.[5–7]

Thanks to the introduction of proper preventive workplace practices and legislative measures, both the morbidity and the mortality of silicosis have been gradually declined throughout time.[5,8]

However, there is a dearth of information concerning silicosis-related interest at the level of scientific community, policy- and educational-makers, media coverage, and new information and communication technologies (ICTs) in terms of scientific production, financial supporting and funding, legislative measures and actions, news reporting and web activities, and public understanding and engagement, respectively. The relationship between science, public engagement and the institutional organisms and bodies is multifaceted and extremely complex.[9] Scientists as “*public communicators*” [10] communicate and interact with journalists, who divulge, simplify and, sometimes, amplify the severity of diseases or health risks.[11] Sensationalism, imbalance and distortion can affect the proper reporting of the news.[12] Further, scientists can be, as well, publicly engaged and can, directly or indirectly, influence normative aspects. Research is supported by policy-makers and other stakeholders, and, as such, scientists have to share their findings, according to the principles of accountability, transparency and responsibility. In conclusion, science is “*inherently political*”.[13]. On the other hand, policy should be based on the latest updated scientific evidences (the so-called “*evidence-based policy making*”), both in the fields of Public and Occupational Health.[14]

In the contemporary society, the widespread use of the Web has revolutionized the interface between science, the public and the media-public interaction. Phenomena such as “*citizen science*” or “*citizen journalism*” (known also as “*collaborative journalism*”) have lead to unprecedented forms of participation and collaboration, in which lay people play “*an active role in the process of collecting*, *reporting*, *analyzing*, *and disseminating news and information*”.[15] In particular, within the framework of e-health or health 2.0, new modalities, strategies, and practices of health care delivery have, indeed, emerged, that exploit, and are supported by, electronic processes and communication, and by a more and more dynamic and interactive information reality. This is the context of the Web 2.0 as a further evolution of the static reality that characterized the Web 1.0: the difference and the distance between webmasters and Web surfers have now become blurred, since the users are, at the same time, consumers and producers (that is to say, *prosumers*) of the Web contents.[16–18]

Within e-health, the patients are at the center of the health care processes, in that they are more involved and informed of the many steps of medical decision making. All the virtual activities carried out by lay people while surfing health-related sites and/or while communicating and sharing their health statuses are known as novel, fast or big data, in that they represent an incredible wealth of data, which are quickly available to researchers.

**Fig 1:**
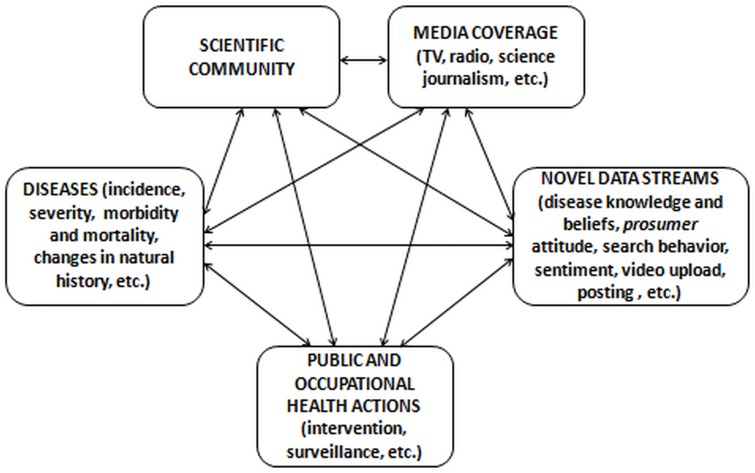
Conceptual framework showing the complex links between scientific community, policy- and education-makers, traditional news coverage and novel data streams, and the public engagement. Adapted and modified from Ref. [19].

Web searches are a complex phenomenon, depending on an array of variables, which include logistic-organizational factors such as the Internet penetration index, user demographics, the searched disease (i.e., incidence, severity, natural history), media coverage, and public and occupational health actions (i.e., preventive interventions, surveillance and legislative measures), among others (Fig 1).[9,19]

In the extant literature, very little is known about the Internet-activities behaviors of patients with chronic-degenerative diseases. Digital tools have been, so far, exploited for capturing interest towards acute diseases[20, 21]. Online tracking systems for early detection of outbreaks are plagued by some methodological issues [22,23] that need to be carefully addressed.[24] The field of behavioral medicine remains, instead, largely unexplored. [25] Traditional surveillance suffers from intrinsic limitations: new and enhanced methods for identification of cases and assessment of disease patterns and trends are, therefore, urgently needed.[24] All over the world, chronic and occupational diseases are under-reported, even if all the countries are legally obliged to use registration systems that provide information on disease-related incidence and prevalence. New digital tools for the epidemiological surveillance of the occupational diseases could be useful to fill in this gap.[26]. Timely and complete reporting is, indeed, fundamental for a successful, physician-based public health surveillance system, especially for the surveillance of occupational health conditions.[27,28]

In particular, there is a dearth of studies focusing on chronic respiratory diseases. Further, findings are contrasting. Delgado and collaborators interviewed 382 subjects with chronic obstructive pulmonary disease (COPD) and they found that 51.6% of the subjects had a computer, 49.7% accessed the Internet, and 13.9% used it to search for information about COPD. The Internet was predominantly accessed by male and younger subjects. Predictors of searching for information about COPD on the Internet were: having a computer, reporting a low dyspnea score, and being of a high socioeconomic level.[29] On the contrary, Duplaga found that, in Poland, 200 patients with COPD reported high levels of acceptance of e-health applications and access to information.[30] Moreover, ICTs seem to enable the design and implementation of a person-centered healthcare delivery for patients suffering from COPD at different steps: from prevention to treatment and rehabilitation.[31–33].

Changes in natural history of diseases or emerging pathogens foster the scientific community to investigate a potential cure or a preventive approach.[34–36] In their turns, scientific production and discoveries trigger news coverage, which, increasing the visibility of articles and contributing to their dissemination and communication, may influence the public behavior, in terms, for example, of interest and digital activities. However, to the best of our knowledge, there are no studies addressing scientific production and web-activities related to occupational diseases.

The present study aims at filling in this gap. We decided to focus on silicosis because it represents a classical occupational disease and also at the light of a renewed interest for this pathology, due to the recently occurred cases clusters.

## Material and Methods

### Big Data analytics

A variety of tools and statistical/computational approaches have been used. They are summarized in Table 1, to which the reader is referred for further details and information.

**Table 1:** Statistical tools and computational approaches used in the current manuscript.

Statistical tools/computational techniques and approaches	Source	Description	Search period
Google Trends	https://www.google.com/trends/	A freely available, online tracking system of Internet hit-search volumes	2004–2015
Wikipedia traffic volumes	http://www.wikipediatrends.com/	A freely available tool that enables to track and monitor traffic volumes related to single Wikipedia pages	2008–2015
Google News	https://news.google.com/	A free news aggregator provided by Google, selecting up-to-date news from thousands of publications	2002–2015
PubMed/MEDLINE	https://www.ncbi.nlm.nih.gov/pubmed/	A free search engine that enables to search among more than 26 million scholarly articles	From inception until 31^th^ December 2015
Google Scholar	https://scholar.google.com/	An open-source web search engine, which indexes scholarly literature including also gray literature and not peer-reviewed material	2004–2015
YouTube	https://www.youtube.com/	A video-sharing website which allows users to freely upload, view, share, and comment on videos	2005–2015
Twitter	https://twitter.com/	A quite popular online social networking service that enables users to send and read short 140-character messages called "tweets"	2006–2015

Google Trends (GT) has been successfully used in a variety of health-related disciplines, investigating the web activities concerning both non-communicable and communicable diseases, for different purposes, ranging from disease monitoring to assessment of public interest towards certain disorders, as recently reviewed by Nuti and collaborators.[37] GT was searched worldwide, using "*silicosis*" as “search topic” (Disease) keyword, in that using this option, all searches related to the query and automatically suggested by GT via its auto-complete service were included.

In the literature Wikitrends has been used to explore information-seeking behavior for epilepsy [38,39] or in order to understand searches concerning scientific and technological terms of broad interest.[9] Wikitrends could reflect academic education.[9]

In the extant literature, Google News has been exploited to investigate coverage regarding smoking cessation campaign or for evaluating the impact of advocacy.[40–42] Twitter has been used to investigate interest toward a wide range of diseases, such as cancer or infectious diseases, among others. It has been exploited to quantitatively assess the effect of advocacy as well [42] or the impact of scientific conferences.[43]

### Data mining

All the above-mentioned tools were mined using an *in-house* script, which facilitated the process of data collection, parsing, handling, processing and normalization.

### Content analysis

Tweets and videos were analyzed according to the following variables: year of publication, number of likes and re-tweets for the tweets, and number of likes and comments for the videos, the nature of the material (institutional or not), if mentioning that silicosis is an occupational preventable disease or not, which occupational activity can cause the silicosis (i.e., fracking, sandblasting, mining, working in textile or automotive industries).

### Statistical analysis

The relationships among scientific production (as measured using PubMed and Google Scholar, in terms of published articles), media coverage (as measured using Google news), search attitude (as measured using GT for lay people and Wikitrends for educational communities, in terms of search and traffic volumes) [9], and *prosumer* behavior (as measured using YouTube and Twitter) related to silicosis were investigated.

All the collected silicosis-related data were modeled as a time series and time-trend was investigated using the Mann-Kendall test and the change-point detection analysis.

Multivariate regression and generalized linear models were performed to understand the silicosis-related interest and activities. More in details, the scientific production was measured using standard bibliometric parameters (countries/territories, number of quotations, impact factor) as well as recently introduced visibility and impact scores (such as Altmetric score). Web activities were assessed according to the number of re-tweets, hash-tags, comments, and likes.

Pearson’s coefficient (r coefficient) was calculated with a commercial software, namely the Statistical Package for Social Science (SPSS) software, version 21.0.0 (IBM Corporation, Armonk, NY, USA). The strength of correlation was measured using the rule of thumb described by Hinkle and coauthors: negligible correlation with r coefficient in the range 0.00–0.30, low correlation with r in the range 0.30–0.50, moderate correlation with r in the range 0.50–0.70, high correlation with r in the range 0.70–0.90, and very high correlation with r in the range 0.90–1.00.[44]

Besides correlational analysis, hierarchical clustering was performed in order to dissect the relationship among the different interest patterns and behaviors (namely, scientific production, news coverage and web-activities). The output of the hierarchical cluster vas visually inspected by means of a dendrogram.

Figures with a p-value <0.05 were considered statistically significant.

## Results

### Scientific production

**Fig 2:**
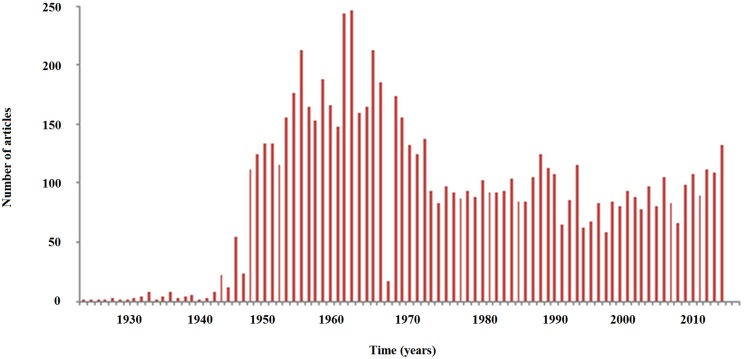
Scientific production related to silicosis throughout years as captured by PubMed in terms of scientific output (number of articles per year).

The scientific production related to silicosis has been globally decreasing from the sixties onwards, with a recent increasing trend in the last decade (with peaks observable in the years 2003, 2005, 2007–2008, 2011, 2013, and 2015) (Fig 2). Some articles have been particular popular, reaching an Altmetric score of 15, being covered by the media and probably contributing to generate tweets and discussion among lay people. Moreover, as far as geospatial localization of silicosis-related scientific production is concerned, this is mainly based in Europe (49.7% of the scientific output) and the USA (24.5% of the scientific output). These two areas together contribute to more than two thirds of the entire scientific production worldwide. China, Russia, South Africa, India, and Turkey contribute to 5.9%, 3.6%, 3.1%, 2.8% and 1.4%, respectively. The Central and South Americas produced the 2.7% of the entire scholarly amount. (Fig 2)

### Google Trends

**Fig 3:**
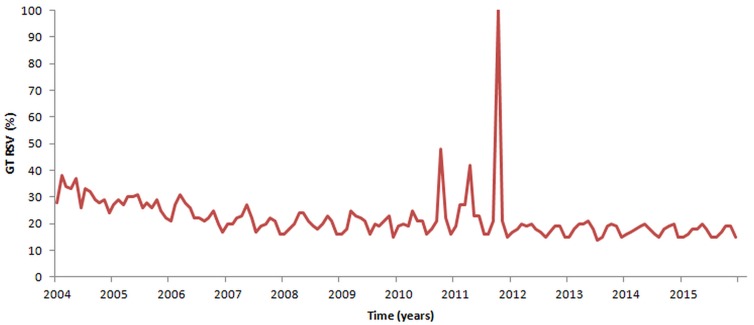
Web queries related to silicosis throughout years as captured by Google Trends.

Since 2004 to 2015, silicosis-related web-activities have remained steady over the years, both for silicosis used as search term or search topic, apart from some peaks occurred in October 2010, April 2011 and October 2011 (Fig 3). According to the Mann-Kendall test and the change-point detection analysis, the search volumes in coincidence of the peaks statistically differed from the web activities in the remaining study period (p<0.0001). Geospatially, the queries were concentrated in the Latin American countries (Chile, Colombia, Peru, Brazil) and in South Africa, less in European countries (Spain, Germany, Italy and Romania) and in Turkey.

Looking at the “most related topics and queries” section, users were particularly interested in a definition of silicosis, its symptoms and clinical presentation, its disabilities and prognosis, and its relationship/co-occurrence with another occupational pneumoconiosis, namely asbestosis (data not shown).

**Fig 4:**
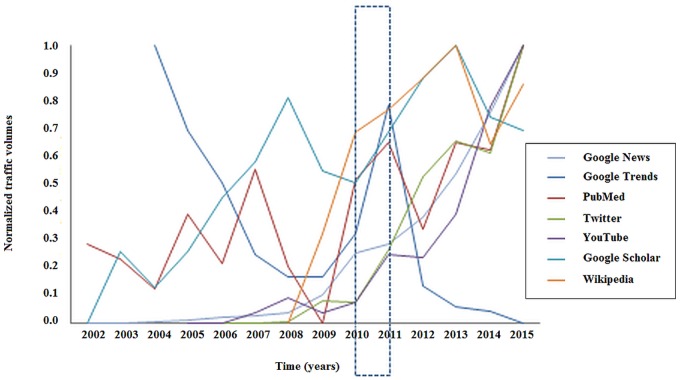
Comparison between scientific production (as captured by Google Scholar and PubMed), news coverage (as captured by Google News), web queries (as captured by Google Trends), access to Wikipedia page and Internet activities (as captured by Twitter and YouTube).

The relationships between GTs and scientific production related to silicosis, in terms of articles published in the literature, and media coverage, verified by looking at the “News” section of GT, YouTube and Twitter, is outlined in Fig 4.

### Wikipedia traffic volumes

**Fig 5:**
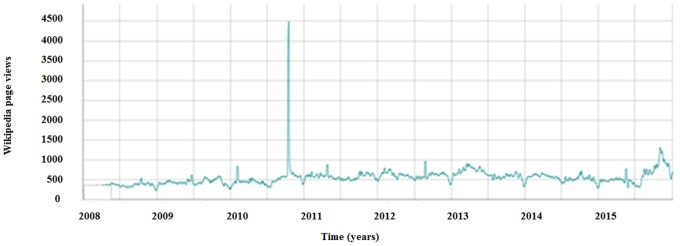
Access to Wikipedia page related silicosis throughout years as captured by Wikitrends.

Searching for Wikipedia page related to silicosis registered a peak in 2010, as captured by Wikitrends (Fig 5). According to the Mann-Kendall test and the change-point detection analysis, the search volumes in coincidence of the peaks statistically differed from the web activities in the remaining study period (p<0.0001). The first five most accessed languages were English (with an average of 470 views per day), Spanish (265 views per day), German (248 views per day), Russian (126 views per day), and French (86 views per day).

### Twitter

A total of 10,879 Tweets were retrieved. The majority of Tweets were written in English, the remaining ones in Spanish, Portuguese, Afrikaans, Hindi/Urdu, Bengalese, Farsi, Chinese, Korean, Russian, French, Albanian, Slovenian, Italian, Norwegian, Danish, Arabic and Swedish. Nearly to 10% of the tweets concerned cases of silicosis occurred in North America, 6.6% in South America (mainly, in Chile), 17.7% in Europe (mainly, in Spain and UK), 52.9% in Africa, 12.6% in Asia (mainly, in India, Pakistan and China) and 0.2% in Oceania.

Approximately 13.4% of tweets of tweets explicitly mentioned that silicosis is caused by exposure to silica dust, 8.6% that is a preventable disease, 18.4% that is an occupational disease, and 0.3% that this condition can lead to death. Some tweets mention historical episodes, including the well-known Hawks Nest Tunnel disaster near Gauley Bridge, West Virginia, USA; others quote normative measures, such as the USA standards to limit worker exposure recently proposed in 2013 and updating those set in 1971, or class actions, such as the Treatment Action Campaign (TACT) or the TB/Silicosis class act promoted by attorney Richard Spoor on 21^st^ December 2012 in South Africa seeking class certification for nearly 17,000 ex-gold miners. Some tweets mention preventive measures, such as technological progress in designing new abrasive blasting procedures with a decreased exposure to silica. Other tweets mention the story of a Chinese gold-miner, He Quangui, recently portrayed in the film “Dying to breathe”. There are references also to art and poetry: the verses of the American poet Muriel Rukeyser and her “Book of the Dead”, commemorating the Hawk's Nest incident. Interestingly, 12.1% of tweets had a personal content, with an increasing trend throughout the years.

**Fig 6:**
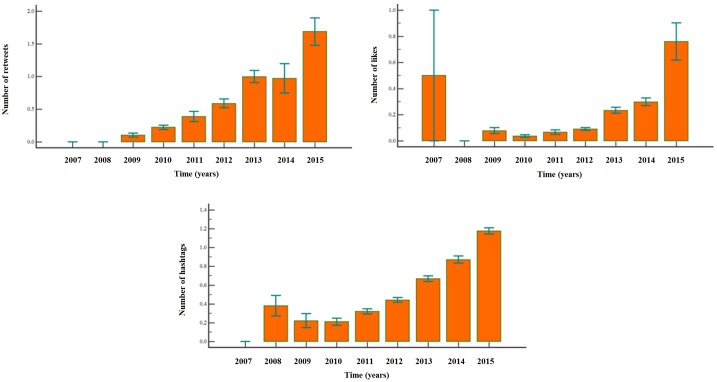
Average number of likes, retweets and hash-tags of tweets concerning silicosis.

**Fig 7:**
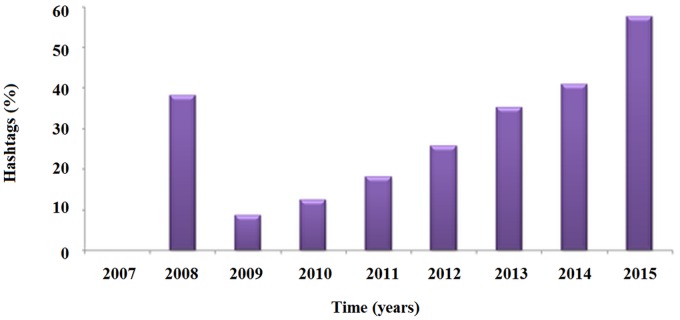
Percentage of hasthags contained in tweets concerning silicosis.

Similarly, likes, re-tweets and number of hash-tags increased significantly throughout time (F = 6.43, p<0.0001; F = 25.49, p<0.0001; and F = 66.02, p<0.0001, respectively), being significantly influenced by place (F = 5.60, p<0.0001; F = 41.86, p<0.0001; F = 27.85, p<0.0001). The personal nature of the tweet correlated with the number of likes, whilst the number of hash-tags was associated with the content of the tweet.(Figs 6 and 7)

### Correlational analysis

Correlation between GT and Google News was negative (r = -0.590, p = 0.0436), as well as between GT and Google Scholar (r = -0.760, p = 0.0041). Correlations between Google News and Google Scholar (r = 0.596, p = 0.0246), between Google News and Twitter (r = 0.961, p<0.0001), between Google News and YouTube (0.974, p<0.0001), between Google News and PubMed (r = 0.800, p = 0.0006), between YouTube and PubMed (r = 0.762, p = 0.0065), between YouTube and Twitter (r = 0.916, p = 0.0002), between Twitter and PubMed (r = 0.725, p = 0.0178), and between Wikipedia and Twitter (r = 0.707, p = 0.0500) were positive. The other correlations were not statistically significant. (Table 2)

**Table 2:** Correlation between scientific production (as captured by Google Scholar and PubMed), news coverage (as captured by Google News), web queries (as captured by Google Trends), access to Wikipedia page and Internet activities (as captured by Twitter and YouTube).

	Normalized Google News	Normalized Google Scholar	Normalized Google Trends	Normalized PubMed	Normalized Twitter	Normalized Wikipedia
**Normalized Google Scholar**	0.596					
	p = 0.0246					
**Normalized Google Trends**	-0.590	-0.760				
	p = 0.0436	p = 0.0041				
**Normalized PubMed**	0.800	0.450	-0.345			
	p = 0.0006	p = 0.1066	p = 0.2728			
**Normalized Twitter**	0.961	0.569	-0.521	0.725		
	p <0.0001	p = 0.0861	p = 0.1229	p = 0.0178		
**Normalized Wikipedia**	0.633	0.304	0.005	0.688	0.707	
	p = 0.0918	p = 0.4638	p = 0.9913	p = 0.0592	p = 0.0500	
**Normalized YouTube**	0.974	0.439	-0.541	0.762	0.916	0.453
	p <0.0001	p = 0.1764	p = 0.0860	p = 0.0065	p = 0.0002	p = 0.2592

### Dendrogram analysis

**Fig 8:**
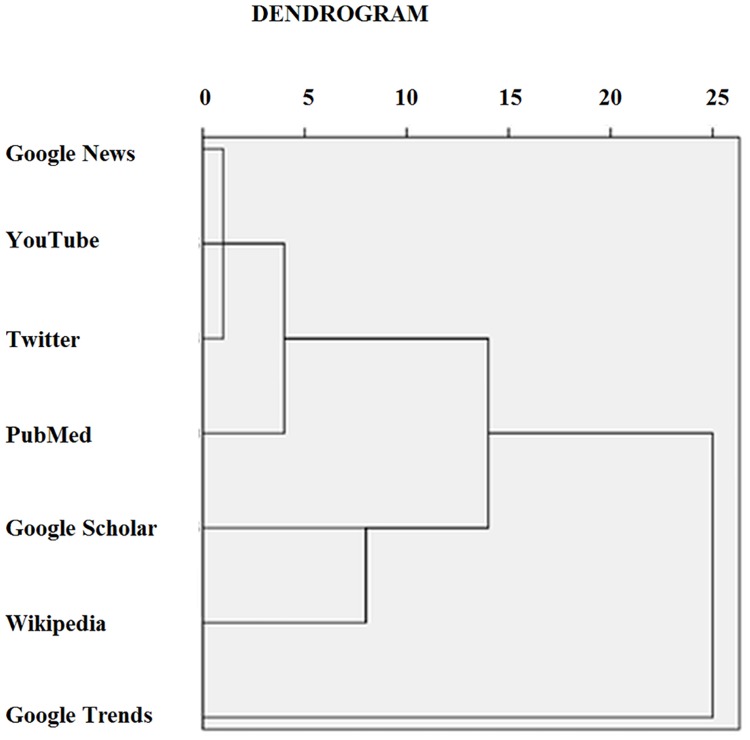
Dendrogram analysis of the relations between scientific production (as captured by Google Scholar and PubMed), news coverage (as captured by Google News), web queries (as captured by Google Trends), access to Wikipedia page and Internet activities (as captured by Twitter and YouTube).

The hierarchical clustering analysis, as visually inspected by means of the dendrogram, showed that scientific production and media coverage clustered together, whilst searching behavior resulted into a separate cluster.(Fig 8)

## Discussion

In the last years, there has been a renewed interest for silicosis at the light of the recently occurred cases clusters characterized by massively advanced clinical symptoms in young adults.[45,46] These silicosis clusters have been reported and investigated by the scientific community, and as well attracted media coverage and called for the need of strict preventive and legislative measures in the workplace.

Scientific interest for silicosis, as investigated by means of PubMed and Google Scholar, increased since 2009 and more remarkably since 2011. This attracted considerable media coverage At the level of lay people, this renewed interest can be captured by GT, which together with Wikitrends, documents superimposable peaks (spikes observable in Figs 3 and 5).

Further, we found that silicosis-related activities were particularly concentrated in the Latin America and South Africa, where silicosis is still exacting a heavy toll [46], possibly reflecting the need of *prosumers* for proper information and prevention measures to avoid the exposure to novel risk-factors.

Thus, our results confirm that both changes in risk exposure and in the natural history of the disease reflect on the related scientific production, media coverage and on search behaviors. We had already noticed this effect in the case of Ebola epidemics [12]. In the case of silicosis, acute massive forms of silicosis have attracted considerable attention and consideration from the public engagement, making evident new form of occupational hazards and exposure to silica and gaps in the workplace safety.

Increase in likes, comments, hash-tags, re-tweets supports an increased interest from the public engagement: the personal nature and content of the web-related activities and material are growing too throughout time with alternating peaks linked to advocacy events [47; 48]. While the half-life of searches and queries is relatively short, the other web-activities, as well as scientific production and news-reporting behaviors, have a longer half-life.

However, there is a knowledge gap. No more than 10% of tweets and posted/uploaded material provide scientific-based, accurate information.

In order to plan and improve adequate preventive programs and large-scale interventions both in public health and occupational field, reliable data are necessary.[27] For this reason, new smart digital systems can be exploited to implement knowledge about the existing or new diseases.[9]

Future studies should investigate online interventions aimed at increasing awareness and knowledge about occupational diseases, in particular about preventive measures that can be adopted in order to safely work without undergoing the risk to exposing to hazardous material.[27]

Our study had some limitations. The major of GT is the lack of detailed information on the method by which Google generates these search data, and the algorithms it employs to analyze them. GT data were available only in the form of relative volume and not as absolute values, enabling further data handling and manipulation. Furthermore, temporal changes in the interface and capabilities of GT over time are not documented, which may lead to variations in the search output and in turn the study findings. Thus, the interpretation of the findings may depend on the chosen time window and geographical area. Furthermore, GT is able to capture only the search behavior of a certain segment of the population—those with Internet access and those using Google rather than other search engines (although Google is the most common search engine). Finally, although some privacy issues exist in using Google Web search data, tracing individuals that conduct online searches when signed into their accounts and recording and analyzing data about users’ characteristics, such as gender and age, intent of web search and “search outcomes” could improve the usefulness of this tool for public health and occupational health education purposes, providing more insights.

## Concluding Remarks

To our knowledge, this study is the first analysis of big data analytics, including media coverage and web search behaviors related to an occupational disease, namely silicosis, carried out both in quantitative and qualitative terms. Very little is known about the level of scientific engagement, news coverage and web-activities related to this occupational disease and how they influence each other. However, ICTs are playing a major role in nowadays society and both occupational physicians and public health policy-makers should be aware of their potentiality.

In the current work, we have shown a renewed interest for silicosis, induced by acute massive clusters and media coverage, produced peaks in search volumes and a marked increase in scientific articles related to this phenomenon. Changes in risk exposures and in the natural history of a disease reflect in changes in search behaviors. These results support adoption of effective preventive and legislative measures which may lead to reduced incidence rate, related scientific production and web activities.

As such, novel data streams could be useful in order to assess the reaction of the public engagement to novel occupational risks, unexpected changes in diseases natural history and the effectiveness of preventive workplace practices and legislative measures adopted to improve evidence based occupational health.[14] Further, occupational clinicians can become aware of the most frequently searched topics by patients and proactively address these concerns and doubts during the medical examination. Institutional bodies and organisms should be more present in the new digital tools to disseminate and communicate accurate information about silicosis

However, this manuscript should be intended as preliminary, exploratory communication, paving the way for further studies.

On the other hand, it must be stressed that we did not formally assess the quality and the correctness of the Web content surfed by the users. Further researches carrying out a formal content analysis of the online material concerning silicosis should be performed.
